# Development and evaluation of Dona, a privacy-preserving donation platform for messaging data from WhatsApp, Facebook, and Instagram

**DOI:** 10.3758/s13428-024-02593-z

**Published:** 2025-02-14

**Authors:** Olya Hakobyan, Paul-Julius Hillmann, Florian Martin, Erwin Böttinger, Hanna Drimalla

**Affiliations:** 1 Center for Cognitive Interaction Technology (CITEC), Bielefeld University, Inspiration 1, Bielefeld, 33619 Germany; 2 Wyss Center for Bio- and Neuroengineering, Geneva, Switzerland; 3 Windreich Department of Artificial Intelligence in Human Health, Icahn School of Medicine at Mount Sinai, New York City, NY USA

**Keywords:** Social interactions, Data donation, Data de-identification, Messaging data, Social network analysis

## Abstract

Social interactions are a fundamental aspect of human life, yet, their objective and naturalistic measurement remains challenging for scientific research. This challenge can be addressed using digital communication data. To this end, we have developed Dona, an open-source platform for donating messaging data from WhatsApp, Facebook, and Instagram. Given the highly sensitive nature of messaging data, we ensure participant privacy through rigorous data minimization. Dona removes all sensitive information on the user side prior to donation, retaining only de-identified meta-data such as message length and timestamps. This paper presents an overview of the platform, a deployment guide, and example use cases. In addition, we evaluate the informativeness of minimized messaging data for studying social interactions with two approaches. First, we conducted a user study in which 85 participants donated their data, received visualizations of their messaging behavior and evaluated the informativeness of this visual feedback. Second, we performed a quantitative analysis using over 1500 donated chats to confirm whether minimized messaging data captures known aspects of human interactions, such as interaction balance, heterogeneity, and burstiness. The results demonstrate that minimized, de-identified messaging data reflects informative interaction features as assessed by both self-reports and objective metrics. In conclusion, Dona is a donation platform well suited for sensitive contexts in which researchers aim to balance participant privacy with the acquisition of objective and informative data on social interactions.

## Introduction

Social interactions are an essential part of human life, influencing both our physical and mental well-being (Holt-Lunstad et al., 2015; Zhang & Fu, 2020). Despite their importance, quantifying social interactions for scientific scrutiny poses a challenge, considering that the social network of a person cannot be simulated in a laboratory setting. A promising avenue for the objective measurement of human interactions is the use of digital communication traces. In particular, a considerable part of human interactions nowadays occurs online, involving social media and messaging services. For instance, in Germany, an estimated 72% of the population used some type of messenger service daily in 2022, with usage rising to 90% among people under 30 (Koch, 2022). These digital interactions are registered on user devices and communication services, creating a useful source of data on longitudinal and authentic human interactions. An emerging approach for obtaining digital traces of social interactions for research is data donation. Data donation makes use of the fact that many smartphone applications and social media platforms allow users to access and save their data, following new data regulations (Ausloos & Veale, 2020), most importantly the General Data Protection Regulation (EU-GDPR, 2018). Consequently, researchers can request data directly from users by asking them to download their data, extract study-relevant information and submit it to the researchers with full consent (Boeschoten et al., 2020). This idea has been implemented in recent studies asking people to donate their data from social media platforms, such as Facebook (Breuer et al., 2022), Instagram (Razi et al., 2022; van Driel et al., 2022), Twitter (Wei et al., 2020) or TikTok (Zannettou et al., 2023). A challenge with these studies is to acquire informative data on social interactions, while simultaneously alleviating the risks associated with sharing sensitive information.

In light of these developments, best practices have been put forward to ensure that data donation is used ethically in research (Ohme & Araujo, 2022). They highlight the need for protecting the privacy of the participants, ensuring informed consent and practicing data minimization, the principle of exclusively collecting data relevant to the research question. For example, data donation tools like Port (Boeschoten et al., 2020), the Data Donation Module (Pfiffner et al., 2024) or ChatDashboard (Kohne & Montag, 2023) allow researchers to create data donation pipelines that ensure only study-relevant information is automatically extracted. While this is an important advancement for privacy-preserving data donation, the burden still falls on researchers to navigate the varying configurations of each tool to carefully manage what data are excluded. The lack of standardized data minimization practices is reflected in the existing studies, in which the exact implementation of data minimization varies widely. For example, many studies interpret data minimization as enabling the participants to preview their data and to choose specific items they wish to share (Zannettou et al., 2023; Breuer et al., 2020). Nevertheless, the collected data are inherently sensitive, even if the participants have previewed it, as plain text messages (van Driel et al., 2022; Razi et al., 2022), comments (Breuer et al., 2022; Zannettou et al., 2023), videos and photos are shared (Razi et al., 2022; van Driel et al., 2022). Researchers often perform de-identification steps, such as hiding or blurring faces (van Driel et al., 2022) or even manually editing sensitive text by going through the shared messages (Razi et al., 2022). Current de-identification methods seem more straightforward for structured data, e.g., when replacing usernames or other sensitive information in well-defined database columns. By contrast, de-identification of sensitive information in the raw data, such as first names or videos remains difficult (Boeschoten et al., 2021). Complete de-identification has been a challenge even in research using comparatively sparse data, such as mobile communication data containing call times and durations (Saramäki et al., 2014; Blondel et al., 2015). Such data often includes identifiable location information (Blondel et al., 2015; Eagle et al., 2009) and contains connected users who can easily be re-identified (Mayer et al., 2016).

While data minimization is a commendable practice, a notable concern is the informativeness of sparse data for studying social interactions. This raises questions about the trade-off between privacy protection and the usefulness of the data for research purposes. Sparse mobile phone communication data has been successfully utilized to study various aspects of social interactions, such as the balance (Kovanen et al., 2010; Wang et al., 2013), heterogeneity (Alshamsi et al., 2016; Miritello et al., 2013), and temporal features of social interactions (Karsai et al., 2018; Aledavood et al., 2018). Nonetheless, it remains unclear whether the interaction patterns observed in previous research on social interactions will generalize to de-identified messaging data.

Here, we present a novel approach for balancing the need for informative data on social interactions with privacy concerns. To this end, we developed Dona, a data donation tool designed for donating data from messaging platforms like WhatsApp, Facebook, and Instagram. To comply with the best practices in the field, we employed *extreme data minimization*, such that only meta-data is donated, without storing any content and identifiable data. De-identification occurs in participant browsers such that the raw data never leaves the participant devices. In the next sections, we present an overview of the platform, offer a deployment guide and describe the details of our implementation. Finally, we present our evaluation study in which the donated messaging data was used to verify whether digital messaging data reflects general aspects of social interactions.

**Fig. 1:**
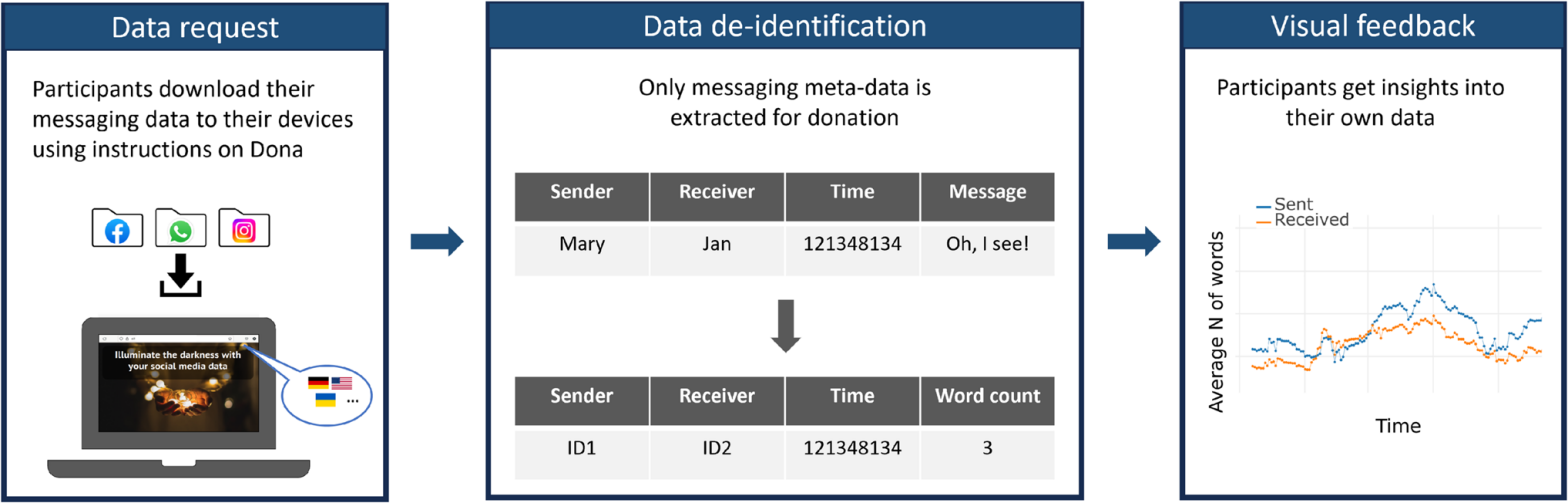
Overview of the donation process with Dona. Data are first requested by the participants from WhatsApp, Facebook, and Instagram (*left*). The participants can choose the platform language from five available options. Once requested, the data files can then be de-identified on the Dona website in the participant browser. Only these de-identified data are submitted for donation (*middle*). To thank the participants, automatically generated visual feedback is provided illustrating their messaging data (*right*)

**Table 1:** Comparison of the messaging platforms for different aspects of the data donation

	WhatsApp	Facebook and Instagram
Device for data export	app-based mobile device	app-based mobile device and computer
Chat export	individual	collective export of all chats
Processing time of data request	immediate	minutes to days
Recommended media export option	without media	low quality
Limiting donation period	on Dona	on the social media platform
Donation file type	.txt or.zip	.zip
Donation of audio messages	no	yes

## The data donation platform Dona

### Overview of the platform

Dona is a web-based, open-source platform for donating messaging data from WhatsApp, Facebook, and Instagram. On the platform web page, the participants first receive information about the goals, incentives, and procedures of data donation. People wishing to participate can then proceed with the donation by providing their explicit consent. The process of data donation consists of three components: request of personal data from WhatsApp, Facebook, or Instagram, data de-identification and visual feedback to thank the participants for their donation (Fig. 1). The platform is currently available in five languages: English, German, Ukrainian, Armenian, and Russian-selected based on our proficiency in these languages.

**Table 2:** Implementation of different aspects of the Dona platform and their management

Component	Implementation	Management
Setup and hosting	Self-hosted server	Researchers
Installation	Docker images	Provided by Dona
Data management	PostgreSQL database	Provided by Dona
Data persistence	Docker volumes	Provided by Dona
Architecture	Play Framework with Scala, JavaScript, HTML, CSS	Provided by Dona
Languages	English, German, Ukrainian, Armenian, Russian	Provided by Dona
Survey	Flexible	Researchers
Dona-survey linking	ID input or output	Provided by Dona

#### Data request

The Dona web page details step-by-step instructions on how to obtain messaging data from WhatsApp, Facebook, and Instagram. Following these instructions, the participants can download files containing their chat history from the messaging platforms to their devices (app-based mobile device or laptop/desktop) and proceed with the donation using the Dona website. While all three platforms provide downloadable files with messaging information, the request process and the formats are somewhat different (see Table 1). In WhatsApp, each chat needs to be exported individually on a mobile device. Once the chats are exported, output files containing the chat history (in.txt or.zip format) are immediately available for download. The processing of data requests by Facebook and Instagram may take several minutes to hours. Once this process is complete, the participants should receive a notification and can download their data. However, as mentioned by Hase et al. (2024), these notifications are not reliable, which is why participants need to check the status themselves. This can be supported by reminder e-mails, which is currently not a feature of Dona. Facebook and Instagram allow downloading information about multiple aspects of a user’s activity, e.g., posts, comments, reactions, messages. Dona currently focuses on donations of messaging data, hence, the participants are asked to limit their data requests to messages and profile information, excluding all other aspects. In case participants do not comply, the surplus data are discarded to ensure that the donation remains anonymous. However, unlike WhatsApp, for which we recommend chat export without media, Facebook and Instagram always export the chats with media. As Dona discards all media other than audio files of voice messages (see next section), we recommend selecting the "low quality" option for the export of media files when requesting data on these platforms. On Facebook and Instagram, the participants can choose the time window for which they are requesting their data. We recommend a period of one year for meaningful longitudinal analysis and manageable file sizes. As WhatsApp does not have a built-in option for limiting the export of data range, we offer this option on Dona.

#### Data de-identification

The participants first import the data files requested from the messaging platforms into the Dona web page. Then, the de-identification process replaces all usernames with pseudonymous IDs and extracts the timestamp and length of each message, resulting in anonymized meta-data containing no plain text (Fig. 1, middle). In addition, for Facebook and Instagram, de-identified information on voice messages is donated including timestamps and length of voice messages in seconds. This is not possible for WhatsApp as media files are not reliably exported. Importantly, the de-identification process is completed locally in the participant browser, as recommended in Boeschoten et al. (2022). Thus, no raw data leaves the device of the participant. The participants can inspect the anonymized data, limit the time period of donation and submit it as a donation. Note that, in case of donations from multiple platforms by the same donor, only the donor can be matched across platforms while there is no way of linking chat partners.

#### Visual feedback

Immediately after data donation, the participants receive visual feedback providing insights into their own messaging behavior (Fig. 1, right). The feedback contains several plots and illustrates three aspects of social interactions: intensity (measured by word count), active hours and response times. When implementing the feedback feature, we took special care to make the visualizations interactive, allowing a self-guided and flexible exploration. For instance, the participants can see how many words they have sent to each of their contacts for specific time periods of interest. They can also download these images to their own devices for future reference. Importantly, the visualizations only show information about the participant’s behavior and the information about contacts is always aggregated. If a time window contains data from only one contact, no information about the received messages is shown as data aggregation would not be possible for this period. This is done for ethical reasons as the other conversation partners have not provided their explicit consent to visualize their data.

We refer the reader to Materials availability for a demonstration video of the platform and hands-on exploration.

### Researcher guide: Platform setup and utilities

Dona allows client-side anonymization and privacy-preserving donation of messaging data, making it particularly suitable for sensitive applications. The platform is freely available to the researchers for use according to the GNU GPLv3.

Table 2 summarizes the infrastructure details relevant for setting up and using Dona. Dona can be installed using files and documentation provided in the project repository (see Code availability). Specifically, we provide Docker images to ensure a seamless and consistent setup of Dona across different machines. These images are a common industry practice and specify all necessary sources and dependencies of the software. For data storage, the relational database management system PostgreSQL is employed. To persist with the data, Docker volumes are used. For the database structure, we refer the reader to Sect. A.1.

**Fig. 2:**
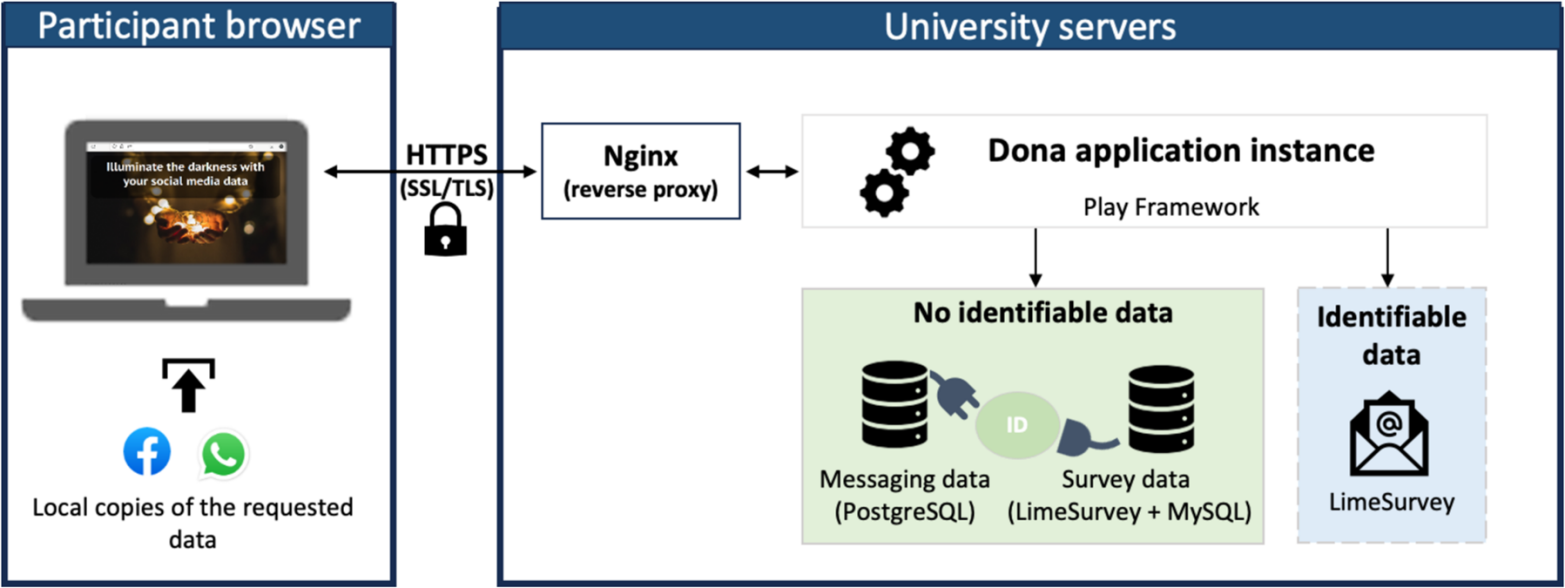
Overview of Dona deployment in our use cases. The participants de-identify the local copies of their data files on the Dona website securely connected to the local devices. The de-identified messaging data are saved in a PostgreSQL database and connected to anonymized survey data via an ID. Identifiable information, such as sign-up e-mails, is temporarily stored in a separate survey. The duration of this storage depends on the relevant administrative or ethical regulations

By installing the provided files following the documentation, Dona can be set up on a server. We recommend using a dedicated virtual machine with access control and encrypted connections to prevent unauthorized access. This step needs to be performed by the researchers wishing to use Dona. Researchers can either host servers themselves or rely on trustworthy cloud computing providers. In addition to setting up Dona as part of an online study, the platform can also be set up on a computer for a lab study. Although Dona already provides extensive functionality, researchers can make further, study-specific adjustments by changing the architectural components within the Play Framework (Table 2) used to build Dona. The platform is offered in English, German, Ukrainian, Armenian, and Russian with a possibility to be extended to other languages.

The donated messaging data can be analyzed alongside other data, such as survey responses. Study-specific questionnaires can be incorporated into the donation process, allowing participants to complete surveys before or after donating their data (see the example implementation below). Additionally, the donated data can be linked to participants’ *existing* data from running or completing studies in two ways. First, researchers can issue a participant-specific ID to be entered before starting the donation process. This ID is then saved as "external_donor_id" (see Fig. 5) to link donations to study data. Second, the researchers can request participants to donate their data using Dona and submit their automatically created ID back to the researchers. While Dona only receives and stores metadata without raw message content, it is crucial to ensure that these linking IDs cannot be connected to identifiable information, such as participant names or e-mails, to maintain complete de-identification. This strict separation of identifiable information and study data is adhered to by Dona and ensures that the participants remain anonymous and non-identifiable throughout the entire pipeline of the data donation.

### Example implementation in sensitive use cases

We have deployed Dona in several studies to examine the digital footprint of significant life events in messaging behavior. The overarching goal is to understand social interaction patterns in response to key life events and their role in mental health. In a typical study, participants fill out psychological questionnaires and provide information about the time period encompassing their data donation, such as important life events, perceived mood and so on. In this context, Dona has also been included in studies focusing on individuals navigating dramatic life events, such as war or other forms of traumatic experiences. All studies mentioned received positive ethics approval, ensuring adherence to ethical standards in research involving human participants.

Figure 2 shows the setup of a typical Dona instance used in the aforementioned studies. The participants interacted with the Dona application using their browsers, while the platform and survey instances were deployed and hosted on university servers using a Linux-powered virtual machine. The connection between the server and the participant browser was established using Nginx with standard secure communication protocols.

The participants request their data following the instructions on the Dona page and submit it to the Dona application. To simplify the process for WhatsApp donations, we ask participants to export only their 5–7 most important chats with close contacts, as exporting individual chats can be tedious. To achieve this, we utilize a name generator method from Perry et al. (2018) by prompting participants to think of people with whom they are most likely to discuss relevant matters. Once the participants request their files and provide them as input on the Dona website, their data gets de-identified in the local browser. Hence, only de-identified messaging data leaves the participant browser to be stored on the university server. From the data donation page, the participants are directed to a LimeSurvey instance to fill in questionnaires. During this redirection process, the ID associated with the donation ("external_donor_id" in Fig. 5) is automatically passed to LimeSurvey to link donation data to the survey data. Identifiable information, such as participant e-mail addresses for compensation, is saved in a separate survey and cannot be linked to either messaging or survey data.

## Online evaluation study

We conducted an online evaluation study to assess the validity of minimized messaging data for the study of social interactions. The study was announced in mailing lists, social networks, on the university campus via flyers and postings, etc. The participants were initially incentivized by the opportunity to see visualizations of their messaging behavior and to enter a raffle for a 50€ voucher. As this strategy resulted in a slow recruitment pace, we changed the compensation to 5€ per participant for 2 months. We invited the potential participants to an online briefing session, in which information about the study and the participation steps were explained. The study, including pilot phases, received ethics approval from Bielefeld University under application number Nr. 2022-194. Our research adhered to the guidelines set forth by the General Data Protection Regulation (GDPR). Further details about the study are available in Sect. B.1.

Eighty-five people participated in this study, in which they donated their messaging data and completed questionnaires. We used the donated data in two ways to examine the informativeness of the messaging data. First, immediately after data donation, the participants received visual feedback on their messaging behavior (see Fig. 1, right) and evaluated their experience using self-reports. The goal was to examine whether the visualizations based on minimized messaging data were informative for the participants. Second, we used the data from all participants to verify that digital messaging data reflected known aspects of social interactions. Here, we used standard measures to summarize the messaging data and to examine whether it reflected characteristics reported in the literature. The approaches are further described below.

### Subjective self-reports

To gather general information about the participants, we asked questions about their sociodemographic profile, including age, gender, education, and employment status. The subjective experience with the visual feedback was assessed using the questionnaires described below.

#### Perceived impact

The perceived impact of the visual feedback was evaluated by a modified version of the *uMARS* questionnaire, section F (Stoyanov et al., 2016). On a seven-point Likert scale, the participants rated whether the visual feedback was informative, improved the understanding of their online interactions, or prompted them to change their messaging behavior, i.e., to increase or decrease the frequency of writing messages.

#### User experience

The short version of the user experience questionnaire (*UEQ-S*) (Schrepp et al., 2017) was used to evaluate the qualities of the visual feedback. We asked four questions on pragmatic (clear, efficient, supportive, easy) and four questions on the hedonic (exciting, interesting, inventive, leading edge) quality of the visual feedback. Answers were given on a slider showing items with opposite meanings at each end (e.g., clear vs confusing). The slider locations were mapped to values between 0 and 100. The scores for hedonic and pragmatic qualities were calculated by averaging the values from the respective questions.

#### Recommending the platform

On a ten-point scale, the participants rated to what extent they agreed with the statement that they would recommend the study to a friend or colleague.

#### Relation to life events

The participants were asked if they noticed changes in their messaging behavior over time and if so, whether they could link those changes to events in their lives.

### Objective features

To verify that the data reflects general aspects of human interactions, we focused on well-documented features of human communication that can be quantified in a relatively straightforward manner: *interaction balance*, *heterogeneity* and *burstiness*. Social ties are generally balanced such that communication partners contribute more or less equally (Kovanen et al., 2010; Wang et al., 2013). Interaction heterogeneity refers to the finding that people show a different level of engagement in interactions with different contacts (Miritello et al., 2013; Alshamsi et al., 2016). Finally, human interactions are bursty in the sense that they often happen in bursts of high activity with intervening periods of silence (Barabási, 2005; Karsai et al., 2018).

#### Interaction balance

We first examined the prevalence of interactive chats in the donated data. Chats were considered interactive if none of the conversation partners contributed more than $90\%$ of the words in a given chat. This was done to exclude artifacts from chats lacking meaningful interactions (e.g., requests from strangers, ads etc.), especially in Facebook donations which contain all chats of the participant. Using this criterion, we identified that the share of the interactive chats was high for both Facebook (*Median* = 0.69, *N* = 7) and WhatsApp donations (*Median* = 1.0, *N* = 78). We used these chats to calculate interaction bias, Gini indices and the temporal heterogeneity for each donation.

For the interaction intensity, we calculated the interaction bias by comparing the number of words sent from the participant to the contacts ($w_{ij}$) and vice versa ($w_{ji}$) using the following formula:

$$\begin{aligned} b_{ij} = 0.5-\frac{w_{ij}}{w_{ij}+w_{ji}}, \end{aligned}$$

where *i* indicates the participant, *j* indicates a contact. This approach is similar to Kovanen et al. (2010) except we subtract the values from 0.5, such that zero indicates no bias.

#### Interaction heterogeneity

We used the Gini index for measuring interaction heterogeneity across contacts using the following formula as in Alshamsi et al. (2016):

1 $$\begin{aligned} g_i= \frac{2\sum _{j=1}^{k}jV_{ij}}{k\sum _{j=1}^{k}V_{ij}}-\frac{k+1}{k}, \end{aligned}$$

where *i* and *j* indicate the indices of the participants and their contacts, respectively, *k* refers to the number of contacts and $V_{ij}$ (sorted in ascending order) refers to the number of words sent from the participant to the corresponding contact.

#### Burstiness

The burstiness metric is calculated based on inter-event times, i.e., how many days have passed between two consecutive interactions. We first identified chats with at least ten interaction days for a meaningful analysis. Then, the coefficient of variation is calculated

2 $$\begin{aligned} r=\frac{\sigma }{\mu }, \end{aligned}$$

where $\sigma$ and $\mu$ refer to the standard deviation and mean of the inter-event times, respectively. We used this coefficient to calculate the burstiness metric, suggested by Goh and Barabási (2008):

3 $$\begin{aligned} B_1 = \frac{r-1}{r+1}. \end{aligned}$$

## Results of the online evaluation study

Eighty-five people participated in the online evaluation study of Dona. From those who filled in sociodemographic information, 42 indicated female, 37 male, and 1 diverse gender identity. Most participants were young ($Median = 25.5$ years old, $SD = 5.7$, $N = 82$), many had completed some form of higher education ($N = 43$) and were employed ($N = 51$ vs $N = 30$ unemployed). Please note that some participants submitted partial data as none of the questions were mandatory.

**Table 3:** General information about the data donations from WhatsApp and Facebook

Donation characteristics		WhatsApp	Facebook
Number of donations		78	7
Number of chats		460 (4.41 % group chats)	1742 (8.95 % group chats)
Number of interactive chats		415 (3.32 % group chats)	1193 (4.54 % group chats)
Chats per person (including group chats)	Median	5.0	251.0
	Mean	5.9	248.86
	SD	2.2	186.26
	Range	5-21	39-551
Group chats per person	Median	1.0	6.0
	Mean	0.94	14.29
	SD	1.03	18.68
	Range	0-4	0-56
Donation timespan (Days)	Median	1956.5	1093.0
	SD	992.21	399.23
	Range	15-4990	163-1095
Donation message count	Median	31k	36k
	SD	43k	52k
	Range	670-188k	1248-166k

Note that the difference in the number of chats from the WhatsApp and Facebook donations stems from the difference in the data request procedures described in Section “Overview of the platform”

**Fig. 3:**
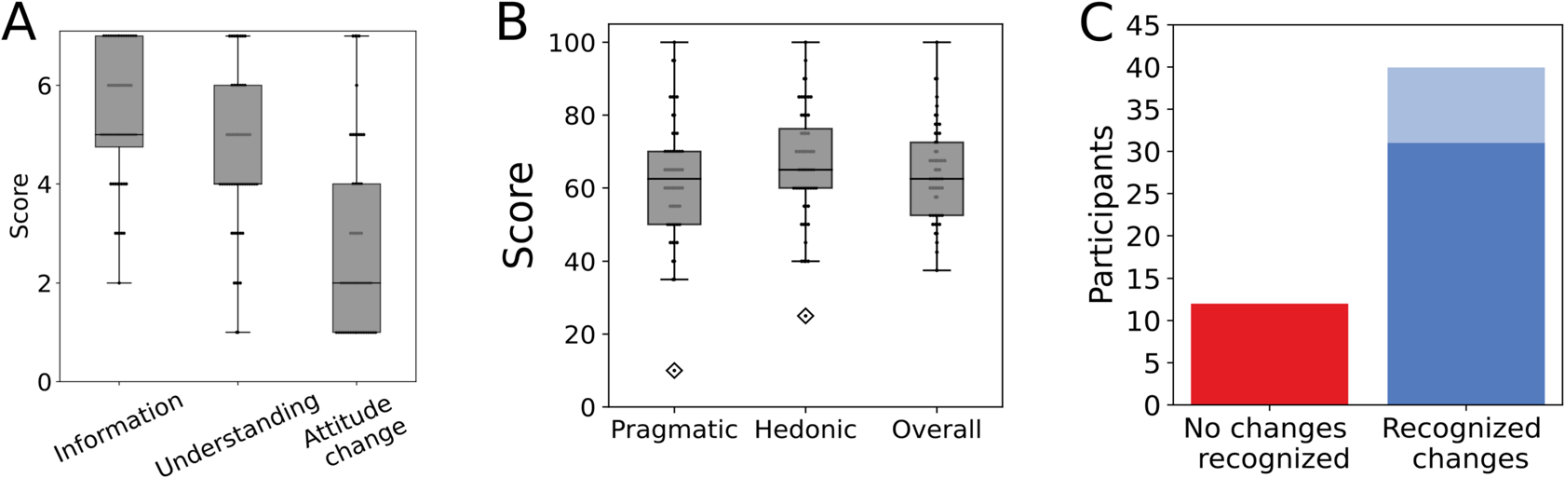
Participant experience with the visual feedback. **A** Perceived impact of the visual feedback regarding informativeness ($N=56$), improved understanding ($N=55$) and intention to change messaging frequency ($N=55$). **B** Scores for the user experience with the visual feedback, separated into pragmatic ($N=56$) and hedonic ($N=56$) qualities. In all box plots, the *box* indicates the quartiles, while the *whiskers* reach 1.5 times the inter-quartile range. The *small dots* show data for individual participants. **C** The participant reports regarding whether they observed changes in their messaging behavior when exploring the visual feedback and whether they could relate these changes to any life events (*dark blue*) or not (*pale blue*)

The participants provided 78 WhatsApp and seven Facebook donations (no participants donated for more than one platform). Since Instagram was integrated to Dona into a later stage of the platform development, no data was collected from this source at the time.

Importantly, WhatsApp donations were restricted to the most important 5–7 chats of a participant (see Sect. “Overview of the platform”). Although we received fewer Facebook donations, they contained a much higher number of chats, since Facebook data contains all chats of a participant. Chats from both sources contained, on average, tens of thousands of messages and spanned a time period of several years, although large variations among the participants were observed (Table 3). The imbalance between the number of WhatsApp and Facebook donations may be due to the prevalence of both platforms, as well as the more cumbersome data download procedure for Facebook. Further work is needed to quantify the influence of both factors.

Finally, the proportion of the group chats was rather small in both data sources, hence no dedicated analysis of the group chats was performed in this work.

### Evaluation of participant self-reports

We evaluated the participant experience by asking a series of questions regarding the perceived impact, provided insights and usability aspects of the visual feedback (details in Sect. “Subjective self-reports”). A subset of participants ($N = 15$) from the pilot phase of the platform deployment was excluded from this analysis to avoid potential biases due to personal connections of some participants to researchers. Their messaging data was included in the quantitative analysis detailed in Sect. “Evaluation of objective features”, as retrospective chat data was not subject to researcher-related biases. From the remaining participants, a total of 56 participants answered the evaluation questions. The highest-rated impact of the visual feedback was its informativeness (Fig. 3A left, $Median = 5.0$, 56) on a seven-point Likert scale). The participants ($N = 55$) reported a median agreement score of 4.0 for whether the visual feedback improved the understanding of their messaging behavior (Fig. 3A middle). Low agreement scores were reported when asked whether the visual feedback would prompt the participants to change their messaging behavior (Fig. 3A right, $Median = 2.0$, $N = 55$), i.e., increase or decrease the frequency at which they write messages. Note that attitude change was not a desired outcome, as the feedback was not designed as an intervention. Regarding general user experience, participants reported moderate scores (Fig. 3B). The scores for the hedonic quality ($Median = 65.0$, $N = 56$, on a scale 0-100) of the user experience were slightly higher than for the pragmatic quality ($Median = 62.5$, $N = 56$). The highest-rated items in the user experience survey were the assessment of how interesting (*Median* = 80.0, $N = 56$) and supportive (*Median* = 80.0, *N* = 56) the feedback was. All other items received similar scores to each other (see Appendix Table 4). Finally, the distribution of agreement scores to the question of how likely the participants were to recommend the study showed a median value of 7.0 from the possible range 1 to 10 ($N=$ 56). These results indicate that the participants generally evaluated the feedback as informative and insightful. However, moderate scores of the overall user experience, specifically of the pragmatic aspects, suggest that the platform should be further improved for a more straightforward participation.

Cronbach’s alpha indicated internal consistency of 0.72 for perceived impact and 0.71 for the UEQ scores.

**Fig. 4:**
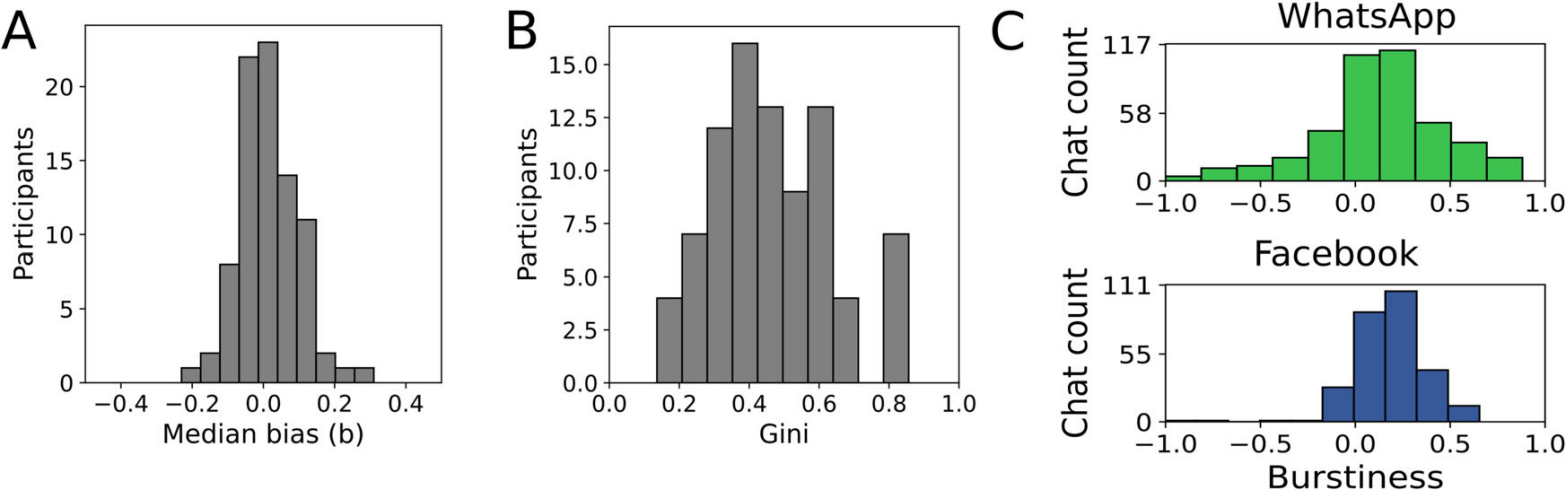
Features of messaging data. **A** The distribution of median bias values for all participants (*N* = 85). **B** Distribution of Gini indices for all participants (*N* = 85). **C** Distribution of burstiness scores for WhatsApp (*N* = 414) and Facebook chats (*N* = 282)

As the visual feedback illustrated participant data behavior over time (see Fig. 1, right for an example), we were further interested in whether the participants would recognize changes in their behavior. Most participants (*N* = 41) recognized changes over time (Fig. 3C). From those participants, the majority (*N* = 31) gave an affirmative answer to the follow-up question of whether they could relate these changes to their life events (Fig. 3C, dark blue bar). Intriguingly, this shows that the participants were able to discern informative changes in their behavior using basic feedback plots on messaging intensity, times, and speed.

### Evaluation of objective features

We conducted a quantitative analysis to confirm that the collected data reflected aspects commonly found in human social interactions, rather than idiosyncrasies specific to digital communication or messaging services. For this purpose, we focused on three interaction features that are well established in the literature and are relatively straightforward to calculate: interaction balance, heterogeneity, and burstiness.

#### Interaction balance

To assess whether the interactions in the donated chats were balanced, i.e., whether the communication partners contributed equally, we first calculated the interaction bias by comparing the number of words sent from a participant to their contacts and vice versa. For this metric, zero indicates no bias, while negative and positive numbers refer to a higher number of words sent by the participant or a contact, respectively (see Sect. “Objective features”). Figure 4A illustrates the distribution of median bias values from all participants. The values tend to cluster around zero, indicating that participants, on average, send a similar number of words as they receive. Although this finding points to generally balanced interactions in our sample, there are participants who either send or receive more words than their contacts (non-zero values in Appendix Fig. 4A). Examples of bias distributions for the chats of individual people can be seen in Appendix Fig. 6. We did not observe any large differences between the platforms in central tendency (WhatsApp: *Median* = 0.01, *N* = 78, Facebook: *Median* = 0.03, *N* = 7) or dispersion (WhatsApp: *SD* = 0.08, Facebook: *SD* = 0.06) of the bias distributions. Our findings confirm the reports in the literature, where largely balanced social interactions co-occur with interactions exhibiting asymmetries either in the participant or contact direction (Kovanen et al., 2010; Wang et al., 2013). These asymmetries can potentially be attributed to different communication styles, messenger use or psychological factors not assessed in this work.

#### Interaction heterogeneity

To measure how equally interactions are distributed among contacts, we calculated the Gini index for each participant (see Sect. “Objective features”). The higher the Gini index is, the more unequally the interactions are distributed, i.e., few contacts receive most of the text sent by the participant. The Gini indices in our sample exhibit individual variations among participants, but tend to cluster around the central values (*Median* = 0.44, *N* = 85) within the possible range of 0 to 1 (Fig. 4B). Hence, our analysis shows that online interactions in the donated chats are heterogeneous without reaching extremes of either complete equality (sending all contacts the same amount of text) or inequality (messaging only one person).

We observed that Facebook donations (*Median* = 0.84, *SD* = 0.02, *N* = 7) exhibit Gini indices almost twice as high as the WhatsApp donations (*Median* = 0.42, $SD = 0.14$, $N = 78$). WhatsApp donations differ from Facebook donations in two ways: a smaller number of contacts ($5-7$) and the selection of the most important contacts (see Sect. “Objective features”). To test how these differences affect the Gini indices, we performed a re-sampling analysis using the Facebook networks. Specifically, we assumed that the chats with the highest word counts may be the most important contacts of the Facebook participants; thus, comparable to the donated WhatsApp chats. Next, for each participant, we sampled either *N* random contacts or the top *N* contacts in terms of word count. Finally, the Gini indices for the sub-networks were calculated, when only these re-sampled contacts (*N*) were considered (details in Appendix C.2). We observed that smaller network size decreases the Gini index (as reported in Deltas (2003)), but the Gini indices are consistently lower when the top contacts are selected (details in Appendix Fig. 7). In an analysis of mobile communication data, the low and high Gini indices were related to psychological variables, such as personality traits (Alshamsi et al., 2016). However, our analysis suggests a more careful interpretation of the Gini index, as it tends to be lower when smaller networks are considered or when contacts are sampled from a similar part of the social network, such as only the top contacts.

#### Burstiness

Another common feature of human social interactions is burstiness, which describes the temporal heterogeneity of interactions, i.e., the distribution of communication events over time (Barabási, 2005; Karsai et al., 2018). Bursty interactions are characterized by a succession of interactions (bursts) followed by periods of silence. To measure the burstiness of the interactions in the donated data, we used a common metric (Sect. “Objective features”), which results in values between $-1$ and 1, with $-1$ indicating regular, 0 random and 1 extremely bursty interactions (see Appendix Fig. 8 for examples). We calculated the burstiness metric for WhatsApp and Facebook data separately. Most WhatsApp (73.43%) and Facebook (87.23%) chats exhibited positive values for the burstiness metric (Fig. 4C). The median burstiness of the pooled 696 chats was 0.17. To test the robustness of our findings, we also estimated the burstiness using a different metric and observed similar results (Appendix Fig. 8). Generally, these findings suggest that the interactions in the messaging data show temporal heterogeneity, indicating communication patterns mostly falling between random and bursty ranges, as reported in the literature (Wu et al., 2010).

## Discussion

We implemented Dona, a novel data donation platform for messaging data from WhatsApp, Facebook, and Instagram. The key feature of our donation platform is its rigorous approach to data de-identification by removing all personal information in participant browsers before data donation. Hence, Dona collects minimized, completely de-identified messaging data containing only anonymized IDs, message timestamps and lengths. We used Dona in an online evaluation study and confirmed that minimized messaging data reflects social interaction features reported in the literature.

In terms of data sparseness, our approach is similar to studies using mobile communication data, in which no communication content is used (Blondel et al., 2015; Saramäki et al., 2014). However, Dona has several advantages compared to these earlier methods. First, our data donation approach obtains direct, explicit consent from participants. This is not possible when researchers obtain the data directly from companies under their terms of service (e.g., from a phone network provider’s call data), as users do not provide direct consent to the use of their data for a specific research purpose (Stier et al., 2020; Ohme et al., 2023). Second, Dona allows the collection of subjective self-assessments or linking of donation data to existing data of the study participants. Some studies using mobile call data have circumvented these issues by distributing mobile phones to study participants (Saramäki et al., 2014). However, this method is costly and not scalable. Third, the way mobile data are acquired and used depends on the data source (e.g., companies or individuals) and is not consistent. Many datasets shared with researchers contain location information (Blondel et al., 2015), while data directly obtained from the study participants is sometimes not sufficiently anonymized. For example, in the study of Saramäki et al. (2014), researchers had access to the phone numbers of both the participants and their contacts.

The consistent and thorough de-identification process is also a feature that distinguishes Dona from other methods of data donation. In particular, previous efforts have focused on first detecting sensitive information in the donated data and then de-identifying it Boeschoten et al. (2021); van Driel et al. (2022); Razi et al. (2022). By contrast, we regard everything as sensitive information and exclusively extract information that does not contain any media or text content. While automatic de-identification is also a feature proposed in other data donation tools, such as Port (Boeschoten et al., 2020), ChatDashboard (Kohne & Montag, 2023) or the Data Donation Module (Pfiffner et al., 2024), there are notable differences between Dona and those platforms. First, platforms like ChatDashboard temporarily store raw data on servers before de-identification is performed, while the raw data never leaves the participant devices when using Dona. This approach makes the application less vulnerable to de-identification attacks or data leakage. While local data extraction in participant browsers is also a feature of Port or the Data Donation module, the de-identification process for these tools is subject to the specifications of researchers using them. By contrast, the de-identification process in Dona is ensured by design, eliminating study-to-study variability and ensuring consistent, standardized data de-identification. This is especially relevant given previous cases showing that identification is possible in ways of which the researchers may not be fully aware (Zimmer, 2010). The *extreme data minimization* approach enhances privacy, making Dona particularly suitable for sensitive applications, such as in healthcare. While minimized data has its limitations, our evaluation study showed that it can still be highly informative.

We showed that the collected data contained longitudinal interactions characterized by the interaction intensities and times from thousands of chats. The results demonstrate that minimized, de-identified messaging data captures relevant aspects of social interactions, as shown by participant assessments and analysis of the collected data. Specifically, participants found the visualizations of their messaging data insightful and informative. Intriguingly, most participants identified changes in their messaging behavior over time and could relate them to specific life events. Future research may integrate targeted questionnaires about the life events associated with such behavior changes. This would help in identifying more precise digital indicators of shifts in social behavior. Quantifying changes in social interactions can be valuable for mental health diagnosis and intervention, as conditions like depression or PTSD are often accompanied by behavioral changes like social withdrawal (Porcelli et al., 2019; Vlachos et al., 2020). In addition to participant assessments, the informativeness of the messaging data was confirmed by our quantitative analysis. In particular, we found that the collected data reflects well-known characteristics from the literature by exhibiting interaction balance (Kovanen et al., 2010; Wang et al., 2013), heterogeneity (Alshamsi et al., 2016) and burstiness (Karsai et al., 2018). While our initial validation confirmed known aspects of social interactions for the digital domain, de-identified messaging data can be combined with more advanced methods like tracking changes over time with temporal analysis, examining the structure of social networks through graph analysis or applying machine learning to uncover hidden patterns in complex data.

Our results show that even minimized data contains rich information about human social interactions when the times and intensities of message exchange between anonymized contacts are analyzed. This is especially important for objectively measuring social interactions as self-reports are often unreliable, particularly when it comes to estimating digital presence (Araujo et al., 2017; Parry et al., 2021; Scharkow, 2016; Ohme et al., 2021). Nevertheless, we are aware that Dona has some limitations. Our data are currently limited to private messages and disregards other channels of communication, such as public posts on social media, media messages and similar information. Future work could extend Dona’s de-identification process to public communication by extracting meta-data from public posts and comments. In that regard, certain types of sentiment analysis would be possible by extracting reaction types from public posts from Facebook or by parsing emojis used in messengers as in ChatDashboard (Kohne et al., 2023). This would further enrich our data, particularly given that sentiment analysis is often used in mental health research (Chancellor & De Choudhury, 2020). Finally, the inclusion of further platforms, such as Telegram, would increase the pool of potential participants, leading to more representative sampling.

Dona faces challenges inherent to data donation, such as incentivizing the participants, navigating the data request process and changes in data export options from the messengers (Kohne & Montag, 2023; van Driel et al., 2022). As Hase et al. (2024) have shown, major platforms do not fully comply with legal requirements regarding data donations and resulting obstacles both increase the effort with which participants can obtain data as well as decrease the ease-of-use for researchers handling such data. Our current web setup does not allow to reliably calculate the dropout rate of the study participants. However, based on our experience, we agree with the general sentiment that participant recruitment is challenging in data donation studies (Pfiffner & Friemel, 2023) and that there is a need for decreasing the burden on the participants (Pfiffner & Friemel, 2023; Silber et al., 2022). While the participants in our online evaluation study found the visualizations of their messaging behavior interesting and insightful, their responses regarding the pragmatic experience show that the platform can be further streamlined. For instance, we provide detailed and transparent information about the donation and the study procedures; however, the amount of information can potentially be overwhelming for the participants. Hence, the presentation of crucial information can be simplified by including explanatory videos. Finally, variations in willingness to donate data across demographic groups can introduce biases (Al Baghal et al., 2020; Ohme et al., 2021; Pfiffner & Friemel, 2023) and pose limitations regarding scientific conclusions. In our study, we only assessed the participant experience with the visual feedback rather than the entire platform. Assessing the complexity of each step during the donation process could provide more insights toward making the platform more user-friendly, especially for less tech-savvy participants. For forthcoming improvements of the aforementioned points, we refer the reader to our project updates at https://mbp-lab.github.io/dona-blog/updates/.

## Summary and conclusions

We proposed an open-source platform for donating completely de-identified messaging data from WhatsApp, Facebook, and Instagram containing only timestamps and word counts. Despite its sparsity, we showed that such data are valuable for identifying informative aspects of social interactions. Visualizations of digital interaction data were informative to the people donating their data, improved the understanding of their messaging behavior and helped them identify changes related to their life events. Furthermore, our quantitative analysis showed that minimized messaging data confirms the aspects associated with general social interactions, such as balance, heterogeneity and burstiness. The privacy-preserving data minimization approach makes Dona particularly suitable for sensitive applications while still ensuring valuable insights through informative social interaction features. When enriched with questionnaire data, minimal messaging data can be used to study human social interactions over time and identify interaction patterns associated with different psychological variables.

## Figures and Tables

**Fig. 5:**
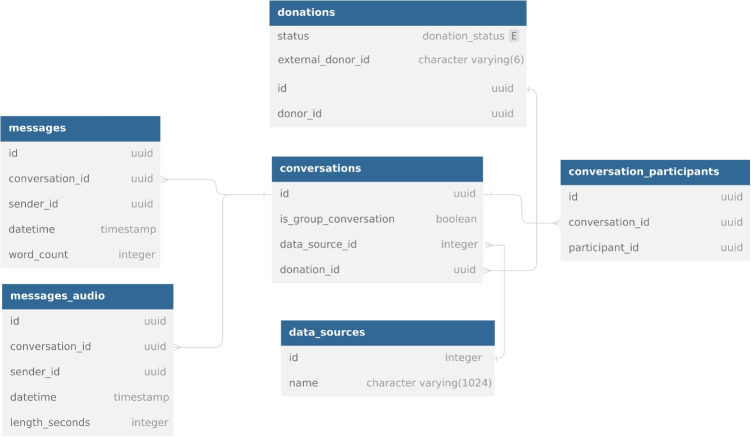
The structure of the relational PostgreSQL database, in which the donated data are organized

**Fig. 6:**
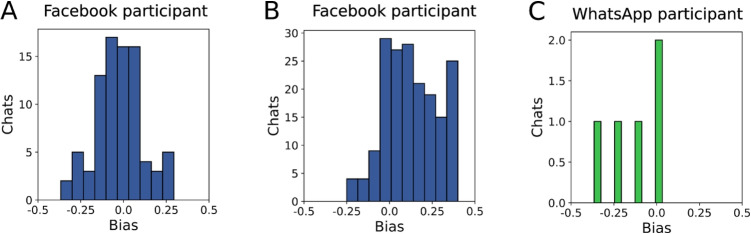
Bias distributions of different participants. **A** Although there are chats with different bias values, the interactions are generally balanced (*Median* = $-$0.02) for this participant. **B**, **C** By contrast, the participants in B and C show lopsided distributions, pointing to a general bias in positive (*Median* = 0.12) and negative directions (*Median* = $-$0.1), respectively

**Fig. 7:**
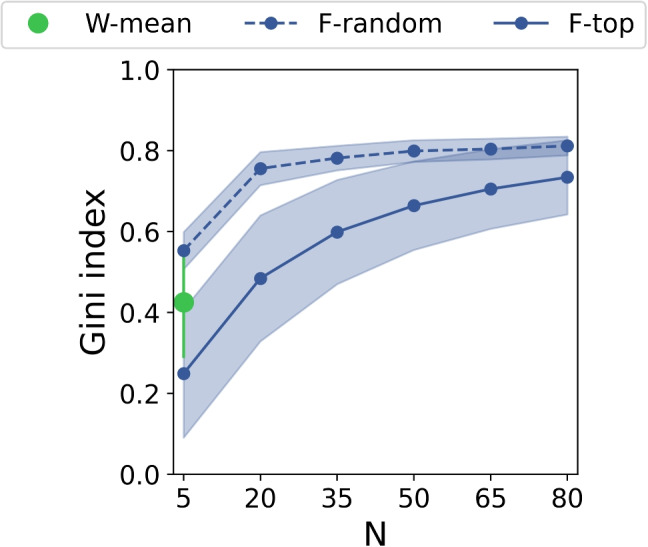
The effect of network size and contact rank on the Gini index. *Shaded regions* indicate the standard deviation across all participants for each value of *N*. The *whiskers* of the WhatsApp data denote standard deviation across all WhatsApp donations

**Fig. 8:**
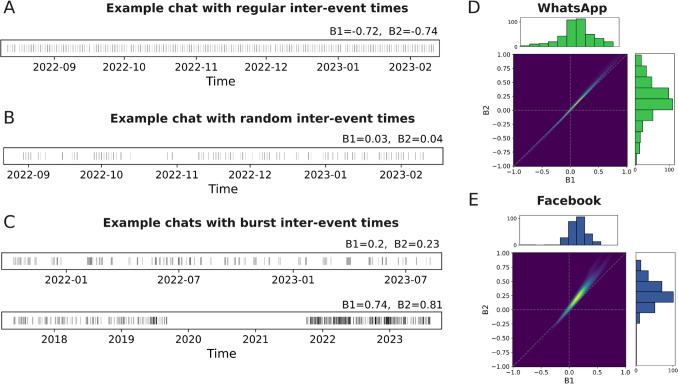
Chat examples from the collected data, exhibiting regular (**A**), random (**B**), or bursty (**C**) interactions. Each *spike* indicates a day in which the participant sent a message. **D**, **E** The burstiness estimates of pooled WhatsApp chats and Facebook chats. The histogram and heatmap axes are aligned

## Data Availability

A demo video of the platform and test files for trying out are available at https://mbp-lab.github.io/dona-blog/. The data from the online study is available in the public data repository at https://zenodo.org/records/12570525.

## Appendix A Details of the donation platform

### A.1 Database structure

The donated messaging data are organized in a PostgreSQL database. Figure 5 shows the structure of the relational database. First, each donation attempt gets a donation ID and its status is recorded. A donation attempt is registered whenever a person accepts the online consent. At this stage, the donation status is set to *pending* and updated to *complete* once the donation is completed, e.g., the de-identified files are submitted to the server.

The *external_donor_id* is the ID used to connect the donated data to other data from the participant, such as survey data outside of Dona. *donor_id* is the ID used to distinguish the data donor from other conversation participants. The donor is automatically determined during the de-identification process. This follows either based on profile information contained in the donation (for Facebook or Instagram) or on the recurring person in all donated chats (WhatsApp). In the table *conversations*, each chat receives its own ID. In addition, the table details whether each chat was a group conversation and the source of the data (WhatsApp, Facebook, or Instagram). The tables *messages* and *messages_audio* contain information about the de-identified text and voice messages, respectively. This includes the length of the messages (word count or length in seconds), sender information, date, time, and the conversation in which the message was sent. Finally, the table *conversation_participants* lists all participant IDs for each conversation.

## Appendix B Details of the online evaluation study

### B.1 Recruitment

We started the online study in February 2023. The recruitment process using online briefing method consisted of four parts: the announcement of the study, the online briefing session, study participation, and, finally, compensation.

The announcement of the study was mainly done through the distribution of flyers - digital and on paper. These flyers showed a QR code that could be scanned and lead to a website where potential participants could sign up for a briefing session, which always took place online as a video conference. Once a participant signed up for such a session, the link to the video conference was sent to them via e-mail.

The briefing session lasted for about 10 min, in which a member of our group introduced the research lab, the study goals, and a demonstration of Dona. This was done following a standardized text as a guideline. Following the presentation, potential participants had the opportunity to pose questions and opt for participation. After the study participation, a link directed them to a form where they could sign up for the 5€ reward. To prevent multiple participations for financial gain, we included a token in the invitation e-mail, which they had to enter during compensation sign-up. This way we could ensure that each token was only compensated once. The tokens and e-mails were not linked to the donation or questionnaire data.

### B.2 Participant experience

The following table illustrates the descriptive statistics for the individual items in the UEQ-S survey. The first four items correspond to the pragmatic qualities, while the last four characterize hedonic qualities.

### B.3 From chat download to browser import

The steps from participants pressing the *download* or *export chat* buttons to importing the file into the Dona platform differ depending on the operating system and software version. Depending on the operating system or software version, participants can either save the file directly to the file system, send it to themselves via e-mail or Bluetooth, or use another app that can handle the file type. Once this is done for all files, participants can use Dona to open the file browser and navigate to the location where all chats are stored. There, they select all the chats at once by long-pressing one chat and tapping the others. The information material on the website aims to facilitate this process.

**Table 4:** Individual UEQ-S item scores for user experience assessment

Quality	Median	Mean	SD
Obstructive|Supportive	80.0	71.43	16.41
Complicated|Easy	60.0	54.64	27.12
Inefficient|Efficient	60.0	65.0	19.73
Confusing|Clear	60.0	58.21	24.86
Boring|Exciting	60.0	65.36	24.93
Not interesting|Interesting	60.0	73.21	22.45
Conventional|Inventive	60.0	65.36	20.53
Usual|Leading edge	60.0	61.43	19.22

## Appendix C Details of the quantitative analysis

### C.1 Interaction balance

We examined how balanced the chats for each participant (Fig. 6) were. To assess the prevalence of such chats in our sample, we inspected the bias distributions of each participant and calculated the median bias of their chats as shown in the main text (Fig. 4A).

### C.2 Interaction heterogeneity

To explore the role of the sample size and contact type on the heterogeneity metric, we also calculated the Gini index using different sub-networks from Facebook donations. In particular, we first identified a maximum network size that would work for all Facebook donations, considering that each donation includes a different number of chats. For that, we calculated the smallest common network size under the constraint that it is larger than 50 to allow a meaningful sampling range. For each Facebook donation, we sampled either *N* random contacts or the top *N* contacts in terms of word count, where *N* in [5,80]. Then we calculated the Gini index when only those contacts were considered. Given the combinatorial nature of selecting sub-networks from the larger network, we opted for a fixed number of repetitions (100) for each *N* while analyzing individual donations. Then, we calculated the average Gini index for a given *N* for each donation and finally, calculated the means across the donations (seven in total).

On average, Facebook sub-networks that consisted of five randomly sampled chats exhibited a lower Gini index (*Mean* = 0.57) than when the full Facebook networks were considered (*Mean* = 0.83), indicating that network size influences the Gini estimates. Compared to random sampling, the sub-networks consisting of the top chats result in even lower Gini indices. As shown in Fig. 7 (dashed vs. solid lines), this pattern is consistent for different values of *N* before the sub-networks gradually increase to the entire network size. This means that sampling from similar parts of the social network (e.g., only top contacts) also influences the Gini index, especially for small networks. The average Gini index from the WhatsApp donations (Fig. 7, green dot) falls between the Gini indices of the random and top sub-networks of the same size from Facebook, indicating a potential interplay of both network size and the parts of the network that is being sampled from.

### C.3 Burstiness

Fig. 8 illustrates example chats with different levels of temporal heterogeneity. Interactions can happen at regular (Fig. 8A) or random intervals, following a Poisson distribution (Fig. 8B). In addition, interactions can be bursty, i.e., occur in quick succession followed by extended periods of no communication (Fig. 8C).

In Fig. 8D and E, *B*1 refers to the burstiness metric used in the main text. $B_1$ is supposed to be susceptible to the number of events (here: interaction days), hence we also used a second burstiness metric *B*2 as defined in Kim and Jo (2016):

4 $$\begin{aligned} B_2 = \frac{\sqrt{n+1}r-\sqrt{n-1}}{(\sqrt{n+1}-2)r+\sqrt{n-1}}, \end{aligned}$$

where *n* refers to the number of events. Both metrics result in values between $-1$ and 1, with $-1$ indicating regular, 0 random and 1 extremely bursty interactions.

## Code Availability

The open-source code of Dona is available at the repository https://github.com/mbp-lab/openDona. The analysis code is available at https://github.com/mbp-lab/dona-brm.
